# The Mid- and Long-Term Consequences After Surgically Treated Lisfranc Injuries: A Case Series and Review of the Literature

**DOI:** 10.7759/cureus.74591

**Published:** 2024-11-27

**Authors:** Laura Fontanella, Joseph M Schwab, Amal Chidda, Moritz Tannast, Angela Seidel

**Affiliations:** 1 Department of Orthopaedics and Traumatology, Ente Ospedaliero Cantonale, Locarno, CHE; 2 Department of Orthopaedics and Traumatology, HFR Fribourg, University Fribourg, Fribourg, CHE; 3 Department of Orthopaedics and Traumatology, Inselspital, University Hospital Bern, Bern, CHE; 4 Departement of Orthopaedic Surgery and Traumatology, HFR Fribourg, University of Fribourg, Fribourg, CHE

**Keywords:** a systematic review, efas score, lisfranc fracture, long-term outcome, sf-12 quality of life scale

## Abstract

Background

Long-term follow-up data are difficult to collect, especially in uncommon foot injuries. Therefore, it is rare to find publications that include patient-reported outcomes. Therefore, a case series and systematic review are provided to evaluate mid- and long-term outcomes.

Methods

Patients operated for a Lisfranc injury in our hospital, between 2010 and 2016, were included. Patients were invited to fill out a combined European Foot and Ankle Score (EFAS) and Short Form (SF-12) questionnaire and to undergo an x-ray of the operated foot. In addition, a systematic review of the literature was performed, and our results were compared with that review.

Results

Fourteen out of 29 patients (48%, four male, 10 female) were seen at a mean follow-up of 8.3 years. Initial Hardcastle & Myerson Classification was: two A (14%); one B1 (7%); 10 B2 (71%); and one C2 (7%). Three patients underwent multiple surgeries: one external fixation followed by definitive stabilization, and two received fasciotomies (14%). The median EFAS score was 15.5, the EFAS sports score was 14, the SF-mental score was 55.73, and the SF-physical score was 48.25. There was substantial variability in outcomes between patients. 12 patients (86%) also underwent a follow-up X-ray exam, demonstrating a mean Kellgren-Lawrence score of 2.7.

In the systematic review, 20 studies and 1052 feet with an average follow-up of 73 months met the inclusion criteria. Most cases underwent K-wire fixation (396 feet, 37.6%). Screw-only fixation was the next most common treatment (306 feet, 29.1%), followed by plate fixation (46 feet, 4.4%). Only three studies present primary arthrodesis in 68 (7.5%) cases. In 47 of the 686 patients (6.8%) with osteosynthesis (open reduction and internal fixation (ORIF)) a secondary definitive arthrodesis of the Lisfranc joint was required later. The mean American Orthopaedic Foot and Ankle Society (AOFAS) score was 75.

Conclusion

Patients need to be informed about the possibility of limitations of activity, including sporting activity. Mid-term follow up studies are manly limited to ORIF. No operative technique shows an advantage in the mid-term.

## Introduction

Lisfranc injuries are lesions to the midfoot at the level of the metatarsal joint. Usually, the mechanism of injury is a forced abduction and plantarflexion. Less commonly this injury can result from a direct trauma. They are uncommon, representing only 0.2% of all fractures [1], yet their social burden is important. In the Netherlands, the cost per injury, including loss of productivity, is estimated to be as high as €17000 in the first six months. Additionally, it is reported that up to 35% of people sustaining this injury will not be able to return to work if they are employed doing either manual labour or military service [2].

Compared to other foot and ankle injuries, Lisfranc injuries are associated with worse outcomes, even when properly managed [3]. Low patient-reported outcome scores are associated with injury severity, demographics, and pre-existing comorbidities. Different treatment options have been proposed, including closed and/or open reduction with percutaneous K-wire fixation [4,5]. Some authors are proponents of cannulated screws rather than K-wires to improve fixation stability [6]. More recently, plate fixation techniques have been proposed [7,8]. These different techniques have been used for Lisfranc fractures and pure ligamentous injuries. In contrast to different fixation techniques, primary arthrodesis (PA) is mainly proposed for pure ligamentous injuries [9]. Yet, it is still unclear which of those treatment methods leads to the best long-term outcome, as results with a follow-up of more than eight years are rarely assessed [3,10]. In addition, no systematic comparison of these studies has been made.

The aim of the project is to determine the results of Lisfranc injuries in a single centre study eight years after injury including 1) patient-reported outcomes, 2) quality of life scores, and 3) progression of osteoarthritis. Furthermore, we compare our results in these three areas with a systematic review of the existing literature. The two hypotheses of the study are that 1) patients with open reduction and internal fixation (ORIF) have a similar outcome compared to PA and 2) the long-term (i.e. greater than four years) fusion rate is less than 10%.

## Materials and methods

This is a retrospective case series level III and a systematic review of the literature.

Patients

We conducted a retrospective review on a consecutive series of patients who underwent surgery for a Lisfranc injury in our hospital between 2010 and 2016. Inclusion criteria were a minimum follow-up of six years and age over 18 years at the time of injury. Patients were included if they had either pure ligamentous Lisfranc dislocation or bony Lisfranc injuries, independent of the type of fixation. Patients with a concomitant injury on the same foot were excluded. In total 29 patients were eligible to participate in this study. A chart review was conducted to assess demographic information as well as to categorize the trauma as high energy or low energy based on the Advanced Trauma Life Support (ATLS) classification (Table 1) [11].

**Table 1:** High energy trauma Definition of a high energy trauma according to the Advanced Trauma Life Support guidelines [12]

Criteria for High Energy Trauma
Fall from height ≥ 3 m or ≥ 3× body length
Car accident
- > 65 km/h
- Vehicle intrusion passenger compartment > 30 cm
- Vehicle rollover
- Passenger ejection from vehicle
- Fatality in same vehicle
Motor or scooter accident > 32 km/h
Car-pedestrian or car-bicycle impact > 8 km/h
Suicide attempt (any)
Crush injury
Direct impact by blunt object either heavy or at high velocity
Penetrating objects (high velocity)

Injuries were classified according to Hardcastle & Myerson [1] and, additionally, open fractures were noted specifically. The Hardcastle-Meyerson classification distinguishes between A: a total incongruent Lisfranc joint, B: a partial incongruent, and C: a divergent Lisfranc joint [1]. It additionally uses a subclassification for further specification. The operative report was reviewed to determine if osteosynthesis or primary arthrodesis was performed. The chart was reviewed for additional surgeries (e.g., fasciotomies, external fixation) and any complications like infection or wound healing problems. Intraoperative non-weight-bearing X-rays and six-week postoperative weight-bearing X-rays were evaluated for the quality of the reduction, specifically joint alignment and the presence of an intra-articular step-off. 

Outcome parameters

**Figure 1:**
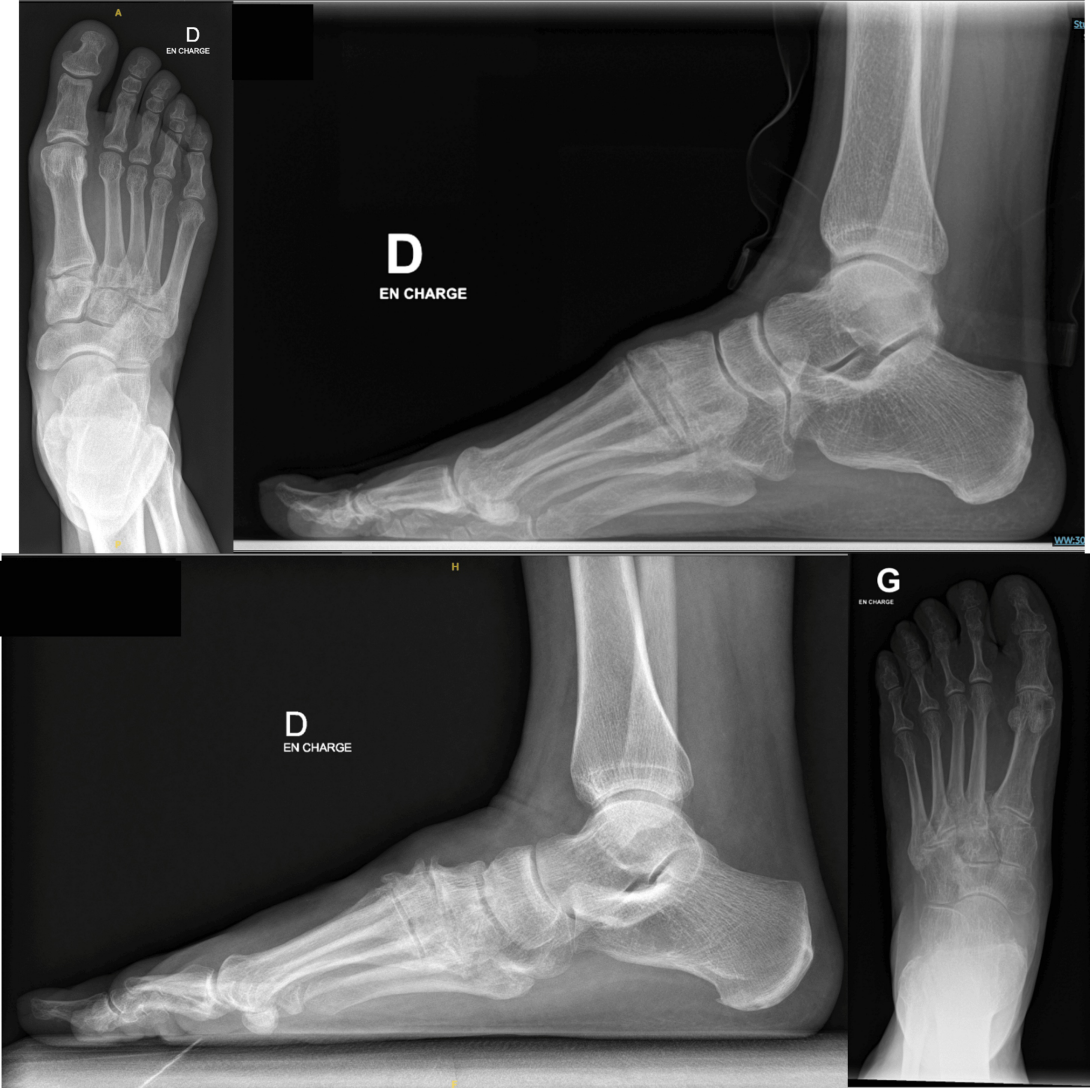
Examples of radiographs showing the Kellgren-Lawrence classification Upper left: Lawrence-Kellgren stage I; Upper right: Lawrence-Kellgren stage II; Lower left: Lawrence-Kellgren stage III; Lower right: Lawrence-Kellgren stage IV [14]

To answer questions 1 and 2, patients were contacted by phone and invited to fill out a combined European Foot and Ankle Society Score (EFAS) and Short form-12 (SF-12) [12] questionnaire on the phone, by post, or in person [13]. All invited patients were a minimum of six years post-injury. The EFAS score is a validated Patient Reported Outcome Measurement (PROM) score of foot and ankle injuries. It is comprised of six questions related to pain and daily activities, and four questions related to sports activities. The SF-12 is a general health status score comprised of 12 questions that assess eight different health domains including physical function, pain, general health and mental health. Each health domain score contributes to a physical component and a mental component score [12]. Finally, to answer question 3, patients who agreed to a clinic evaluation were given x-rays to evaluate post-traumatic arthrosis based on the Kellgren-Lawrence score (Table 2 and Figure 1) [14]. 

**Table 2:** Kellgren-Lawrence classification The Kellgren-Lawrence classification which was used to analyze the degree of osteoarthritis is the follow-up radiographs [14]

	radiographic findings
Grade 0	definite absence of x-ray changes of osteoarthritis
Grade 1	doubtful joint space narrowing and possible osteophytic lipping
Grade 2	definite osteophytes and possible joint space narrowing
Grade 3	moderate multiple osteophytes, definite narrowing of joint space, some sclerosis and possible deformity of bone ends
Grade 4	large osteophytes, marked narrowing of joint space, severe sclerosis and definite deformity of bone ends

Due to the limited number of patients in the study only descriptive statistics have been performed and reported. RStudio (The R Foundation for Statistical Computing, Vienna, Austria) was used for statistical analysis and reporting.

Systematic review

**Figure 2:**
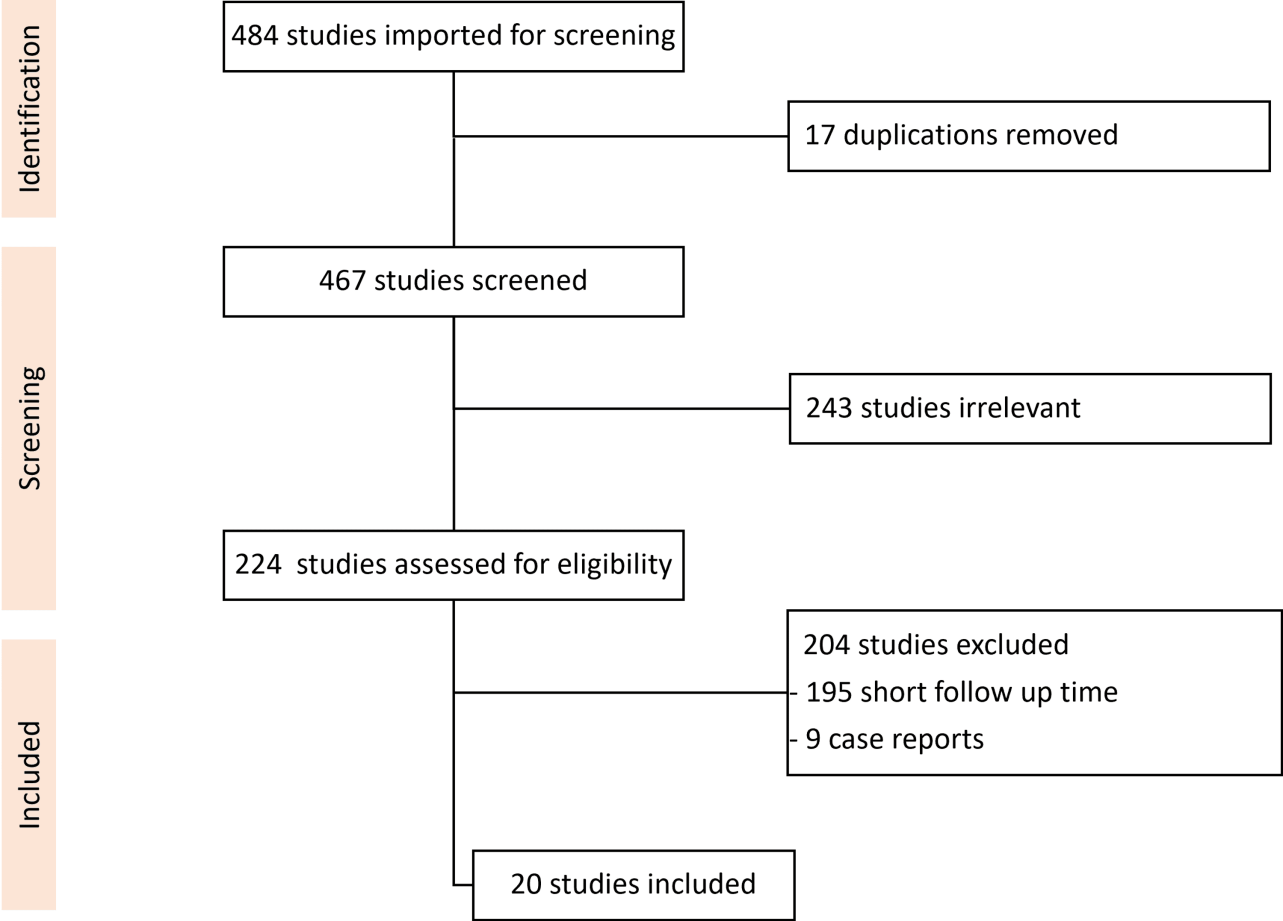
PRISMA flowchart of the systematic review PRISMA: Preferred Reporting Items for Systematic Reviews and Meta-Analyses

A systematic review was conducted according to the Preferred Reporting Items for Systematic Reviews and Meta-Analyses (PRISMA) guidelines. Pubmed, Google Scholar and Cochrane Reviews were searched with the following keywords: “Lisfranc” or “tarsometatarsal fracture” and “outcome” or “dislocation” or “subluxation”. Papers published from 1950 until August 2024 were included. Papers found in the reference list of the relevant articles were also included. The total number of citations found was 484 (Figure 2).

Inclusion criteria were clinical studies of at least 10 adult patients reporting a follow-up of at least four years after the injury. Full-text articles, independent of the language, were included. 20 studies fulfilled the inclusion criteria (Table 3).

**Table 3:** Systematic review Summary of the systematic review of studies with a mean follow-up of more than 48 months compared to our patient population. ORIF: Open reduction and internal fixation; PA: Primary Arthrodesis; AOFAS: American Orthopaedic Foot And Ankle Society Score, si: superficial infection, di: deep infection; OA: Osteoarthritis; na: not available; MFA: Musculoskeletal Function Assessment; SMFA: Short Musculoskeletal Function Assessment;  FFI: Foot Function Index; BFS: British Foot Score; SF-12 PCS: Short Form-12 physical component.

Author	Year of publication	Follow up rate (%)	Number of feet (at follow up)	Age (mean)	Periode of treatment	Hardcastle & Myerson Classification					ORIF / PA in %	Type of fixation				Follow-up in month (mean)	Complication	Number of arthrodesis	Follow-up in month (mean)	Results mean AOFAS	Results
																						A	B1	B2	C1	C2	K-wire	screws	plates	others
																						Buda [15]	2018	na	217	45	1991-2016	7	6	61	1	4	75/25	217				0	7 si, 4 di	11	62	Na	na
																						Demirkale [16]	2013	84	32	35	2004-2009	11	8		13		100/0				screw (+- K-wire for lateral column)	0	5 skin grafts	na	55	59	43.8
																						Dubois-Ferriere [17]	2016	48	61	38	1988-2009	16	27		18		82/18				screws + K-wire	na	1 i	4	130	79	symptomatic OA 54.1%;
																						Eceviz [18]	2021	70	62	38	2007-2017	6	5	41	3	7	95/5		62			7	na	na	57	75	
						Garcia-Renedo [19]	2012	86	83	38		1993-2008	17	25	34							7		76/0	63			20 no fixation	24	3 si,, 1di 2 skin graft, 1 vascular ; 1 pseudoarthrosis	7	102	69	
Kuo [20]	2000	52	48	39	1990-1997	15 ligamentous, 33 mixed					100/0		48			13	2 skin graft, 2 flape skin coverage	na	52	77	
Lau [7]	2016	53	50	na	2006-2013	8	26	12	1	3	100/0		14	19	17 combination	10	6 i	0	58	66	
MacMahon [21]	2016	83	38	32	2006-2014	16% ligamentous, 84% mixed					100/0				screws + K-wire	na	---	1	62	na	97%satisfied
Marin-Pena [22]	2012	91	32	32	1993-2001	11	3	15	3		75/0	24			8 no fixation	na	Na	na	180	91	
Miswan [23]	2011	na	34	30	2000-2006	4	5	14	3	8	100/0	26	5		3 combination	26	Na	1	48	na	BFS: 16 to 25
Modrego [5]	2002	na	25	42	1980-1996	7	9		3		100/0	25				na	2 skin graft	2	84	na	OA absent 4, moderate 18, severe 3
Nithyanatha [24]	2011	59	13	36	1999-2005	3	2	6	1		100/0	13				100	9 split skin graft, 1 osteomyelitis	10	56	82	
Perez-Blanco [4]	1988	91	29	34	1991-1986	divergent:3 ; homolat:5; partial lat:14; partial med 7					79/0	23			5 fixation	na	1si, 3 skin necrosis, 1 amputation	na	74	na	Hardcaste: good: 20; fair: 5; poor: 3
Perugia [6]	2003	86	42	38	1994-1999	14	17		11		100/0		42			0	na	na	58	81	
Poulsen [25]	2023	100	10	34	2011-2015	na					mixed				mixed	0	Na	na	70	91	
Prasla [26]	2024	na	146	40	2007-2012	13	3	129	1		100/0		120	4	33 suture button	na	3 i, 3 wound dehiscence	6	60	na	
Sinkler [3]	2023	36	46	40	2010-2018	na					100/0				multiple	11	na	na	104	na	FFI 35.9
van Koperen [8]	2016	72	34	44	2000-2013	column: medial 28, central: 28, lateral: 22					100/0	5	3	21	5 combination	12	3 i	2	49	75	
Walley [27]	2021	42	19	42	2005-2014	2 pure ligamentous					100/0		14	2	3 combination	0	---	2	75	na	
Walsh [28]	2023	na	31	50	2010-2018	1	28		2		100/0				42% staples, 48% staples + screw, 9% stable + others	na	1 i	1	48	78	FFI 76.8
Our		50	14	50	210-2016	2	1	10	0	1	93/7	6	2	6		14	2 wound	0	99	na	

The study was approved by the ethical committee “CER-VD” (study ID “2021-01120”).

## Results

Twenty-nine patients met the inclusion criteria. Of those 29, 31% (nine patients) were not able to be contacted after three attempts each, 14% (four patients) declined participation, and 7% (two patients) had died. This left 14 out of 29 patients (48%, four males, 10 females) who completed the questionnaire at a mean follow-up of 8.3 years (Table 4).

**Table 4:** Case Series Detailed demographics and outcome parameters of the patients included in the case series. EFAS: European Foot and Ankle Society, SF-12: Short Form 12 questionnaire; ORIF: open reduction and internal fixation; K-wire: Kirschner wire; na: not available *patients with foot compartment syndrome;

Age at Injury	Gender	Mechanism of Injury	Type of Injury (classification)	Implant	ORIF / Arthrodesis	Complications	EFAS score	EFAS sport	SF-12 mental	SF-12 physical	Arthroses
48	Female	High	A lateral	K-wires	ORIF	`---	6	na	61,54	34,46	IV
44	Man	High	B1 Medial	Bridging Plate	ORIF	---	24	16	60,75	56,57	I
52	Man	High	A lateral	K-wires	ORIF	Skin necrosis	22	na	58,64	48,65	na
53	Female	High	B2 Partial Lateral	K-wires	ORIF		24	14	57,19	53,07	II
48	Female	High	C2 Total	K-wires	ORIF	---	15	na	60,66	43,21	III
52	Female	Low	B2 Partial Lateral	Plates	ORIF	--	22	16	50,75	56,42	I
84	Female	Low	B2 Partial Lateral	Bridging Plate	Arthrodesis		22	na	57,81	55,24	III
27	Female	High	B2 Partial Lateral	Bridging Plate	ORIF	*	10	na	37,36	34,48	III
45	Female	Low	B2 Partial Lateral	Screws	ORIF	--	21	16	53,89	52,09	na
58	Female	High	B2 Partial Lateral	Bridging Plate	ORIF	delayed wound healing	16	10	56,86	50,40	II
60	Female	Low	B2 Partial Lateral	Bridging Plate	ORIF	---	6	na	53,12	27,93	III
32	Female	High	B2 Partial Lateral	Screws	ORIF	--	15	9	54,60	41,69	IV
45	Man	Low	B2 Partial Lateral	K-wires	ORIF	--	9	4	53,64	47,93	III
57	Man	High	B2 Partial Lateral	Bridging Plate	ORIF	*	3	na	20,19	24,63	IV

Two of them declined the follow-up radiograph but agreed to complete the questionnaire. The mean age at the time of injury for this group of 14 patients was 50.4 years. 35% (five patients) were smokers and 7% (one patient) had a diagnosis of diabetes. The majority of the analyzed patients (n=10) presented Hardcastle & Myerson [1] B2, then two A, one B1, and one C2. 64% (nine patients) sustained the injury during high-energy trauma and the other 36% (five patients) had sustained low-energy trauma.

One patient (7%) underwent temporary treatment with external fixation for 13 days prior to definitive fixation. Two patients underwent fasciotomies prior to definitive stabilization due to concern for foot compartment syndrome. Thirteen patients (92.8%) underwent osteosynthesis and one patient (7%) underwent primary arthrodesis of the Lisfranc joint. Osteosynthesis included K-wire-only fixation in five patients, screw fixation in two patients and bridge plating in five patients. Two patients presented with an open injury, one of whom developed postoperative skin necrosis.

Analysis of the immediate post-operative radiographs revealed five patients (35.7%) with anatomic reduction, six patients (42.9%) with an intra-articular step less than 1 mm, and three patients (21.4%) with an intra-articular step of 1 mm or greater.

**Figure 3:**
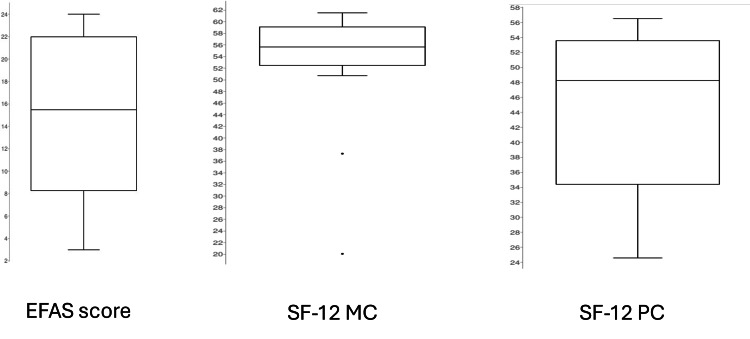
Outcome: Case series Box Blots of the outcome of our patient population at a mean of 8.3 years of follow-up. The figures show a large variation in functional outcomes after this injury. Left: EFAS score: Median 15.5; Min 3; Max 24; First quartile 8.25; Third quartile 22; Interquartile Range 13.75; Outliers none. Middle: SF-Mental Component: Median 55.7; Min 20.1; Max 61.5; First quartile 52.5; Third quartile 59.1; Interquartile Range 6.6; Outliers 20.1, 37.3. Right: SF-Physical Component: Median 48.25; Min 24.6; Max 56.5; First quartile 34.4; Third quartile 53.55; Interquartile Range 19.15; Outliers none. EFAS: European Foot and Ankle Society, SF- 12: Short Form 12 Questionnaire

The median EFAS score was 15.5 (range 3 - 24) and the median EFAS sports score was 14.0 for the 50% of patients who reported performing sports regularly. For the SF-12, the median Mental Component Score (MCS) was 55.73 (range 20.2 - 61.5) and the median Physical Component Score (PCS) was 48.25 (range 24.6 - 56.5) (Figure 3).

Twelve patients underwent follow-up radiographs, and the distribution of Kellegren-Lawrence grades of osteoarthritis was three patients (25%) with Level IV, five patients (41.7%) with Level III, two patients (16.7%) with Level II, and two patients (16.7%) with Level I.

Systematic review

In the systematic review, 20 studies met the inclusion criteria. In total 1052 feet were treated. The most used injury classification (13 of 20 studies: 65%; 683 of 1052 feet:65%) was the Hardcastle & Myerson Classification. There were 118 (17%) type A, 476 (70%) type B, and 89 (13%) type C injuries. K-wire fixation (n=396, 37.6%) was the most common treatment followed by screw-only fixation (n=306; 29.1%) and plate fixation (n=46, 4.4%). Combination fixation or alternative fixation methods were performed in 302 cases (28.7%). No studies reported flexible fixation techniques such as suture button fixation. Only three studies (15%) included primary arthrodesis (n=68, 6.5%). Finally, in 47 (6.8%) of the 686 cases that underwent osteosynthesis, a secondary definitive arthrodesis of the Lisfranc joint was later undertaken. Eight studies did not provide this information at follow-up.

Follow-up ranged from 48 to 180 months (mean 73 months). The most frequently used primary outcome measure was the AOFAS score in 10 of the studies (Table 3). The median score was 75 with a range from 59 to 91. No risk factors for worse outcomes were identified. 

## Discussion

This paper presents outcomes data from 14 patients with a Lisfranc injury, with a mean follow-up of 8.3 years. It shows the importance of the injury in activities of daily living and sporting activities, as assessed by patient-reported outcome measures. Based on our systematic review there are few studies of this injury whose follow-up is longer than eight years. 

American Orthopaedic Foot and Ankle Society (AOFAS) score

The most frequently used outcome score was the AOFAS score, which helped facilitate comparison between studies, but remains a non-validated scoring tool [29]. An AOFAS score between 75 and 94 indicates a good outcome. The median score in our systematic review was 75 (59 - 91). In 2012 Marin-Pena published a case series of 32 patients who mainly underwent K-wire fixation for their Lisfranc injury [22]. This study is the longest reported follow-up at a mean of 180 months. In their series, the mean AOFAS score at the latest follow-up was 91, which is the highest reported for a series of Lisfranc injuries. We find this interesting as our experience indicates that outcomes from this injury worsen over time. Since radiographic outcomes in this study were “fair” in 28% of the cases, the authors concluded that radiographic outcomes do not correlate with clinical results. Their clinical results, however, could not be reproduced. Dubois-Ferriere et al followed a cohort of 61 patients with Lisfranc injuries (almost double the size of the previous study) for a mean follow-up of 130 months and reported a mean AOFAS score of 79 and an osteoarthritis rate of 72% [17]. In these two studies, which represent the only studies with a follow-up over 10 years, the AOFAS score differs by 12 points although both showed a high rate of osteoarthritis. In our systematic review, we did not identify an obvious correlation between the AOFAS score and the duration of reported follow-up.

Other patient-reported outcome scores

In our patient cohort, we used EFAS as our primary patient-reported outcome score. EFAS is divided into two sections, one assessing regular activities and one focusing on sport. No other study in the systematic review used EFAS to evaluate outcomes in Lisfranc injuries. We chose to use EFAS for two reasons. First, EFAS is validated in multiple languages and our patient population is multilingual. Second, we were interested in capturing data regarding sporting activity following this injury since that type of activity is common in our local population. In our cohort, EFAS sports scores showed that half of our patients were not participating in sports regularly following their injury. This may also be age-related as the mean age of our cohort was approximately 50 at the time of injury and almost 60 at the time of final follow-up.

Dubois-Ferriere et al. assessed the FFI (Foot Function Index) as their patient-reported outcome score, which reports pain with different activities with lower scores correlating to less pain. They showed persisting pain during daily activities with a mean FFI of 16.9 [17], which is similar to the study by Sinker et al. who reported persistent limitation and a mean FFI of 36 [3]. While this is similar to our results, the average age of patients in the systematic review was 40 at the time of injury. While age may be a factor in sporting activity, the inability to participate in sport may also be a consequence of the injury. McMahon et al. analysed the difficulty in different sporting activities preoperatively compared to postoperatively in a patient cohort with a mean age of 32 at the time of injury. They found that 97% of their patient cohort was active in sports preoperatively, and of those, 65% returned to at least their preoperative physical activity participation levels [21].

Quality of life

The SF-12 score is a frequently used quality of life score which can be used across a variety of pathologies. In the “normal” German population the mean SF-12 physical component is 49.6 and the mental component 52.3 [12]. In our study population, the physical component (mean 44.9) was slightly lower than “normal” but the mental component (mean 52.6) was no different. Our results are in line with the study by MacMahon et al. who also reported on quality of life in their cohort [21]. Using the Foot and Ankle Outcome Score Quality of Life subscore (FAOS QOL) they reported a slightly reduced quality of life score (75.5) compared to the normal population (79.9) [30].

Radiographic outcome

Post-traumatic osteoarthritis after osteosynthesis for Lisfranc injury is a frequent topic of discussion since while the radiographic incidence can be high, this has not correlated with symptoms. It is reported that up to 72.1% of patients show osteoarthritic findings on radiographs following osteosynthesis, but only 54.1% of those patients are symptomatic [17]. In addition, the reported rate of secondary arthrodesis in patients undergoing osteosynthesis is 7% [17]. Our systematic review also shows a low rate of secondary arthrodesis at 7% (47 of 686 patients), and a combined 15.3% (115 of 754 patients) rate of both primary and secondary arthrodesis. Long-term follow-up for primary arthrodesis of Lisfranc injuries is rare. Given the low rate of conversion to secondary arthrodesis, one should approach the decision to proceed with primary arthrodesis carefully.

Limitations of this study include the low follow-up rate of 50%. It is slightly lower than the average of the systematic review which showed 70% in 15 of the studies. This can unfortunately be a common problem for studies with long-term follow-up. We have included the relevant data on our entire cohort for analysis, but we realize this data is not complete. In addition, there are inherent limitations of a retrospective study over a long-term period such as multiple surgeons being involved, changing indications, etc. Larger prospective studies are needed to determine risk factors for poor long-term outcomes. As this injury is relatively rare, a multicenter approach might be required to obtain the greatest amount of high-quality, long-term follow-up data.

## Conclusions

The analysis conducted on our data confirms the results of available literature about Lisfranc injuries. Patients need to be informed about the possibility of activity limitations including sporting activity. As mid-term follow-up of primary arthrodesis after Lisfranc injuries is rare, future research is needed to provide clear indications for treatment with primary arthrodesis versus osteosynthesis.

## References

[1] Hardcastle PH, Reschauer R, Kutscha-Lissberg E, Schoffmann W (1982). Injuries to the tarsometatarsal joint. Incidence, classification and treatment. J Bone Joint Surg Br.

[2] Hawkinson MP, Tennent DJ, Belisle J, Osborn P (2017). Outcomes of Lisfranc injuries in an active duty military population. Foot Ankle Int.

[3] Sinkler MA, Benedick A, Kavanagh M, Vallier HA (2023). Functional outcomes after high-energy Lisfranc injuries. Foot Ankle Int.

[4] Pérez Blanco R, Rodríguez Merchán C, Canosa Sevillano R, Munuera Martínez L (1988). Tarsometatarsal fractures and dislocations. J Orthop Trauma.

[5] Modrego FJ, García-Alvarez F, Bueno AL, Palanca D, Seral F (2002). Results of the surgical treatment of Lisfranc fracture-dislocations. Chir Organi Mov.

[6] Perugia D, Basile A, Battaglia A, Stopponi M, De Simeonibus AU (2003). Fracture dislocations of Lisfranc's joint treated with closed reduction and percutaneous fixation. Int Orthop.

[7] Lau S, Howells N, Millar M, De Villiers D, Joseph S, Oppy A (2016). Plates, screws, or combination? Radiologic outcomes after lisfranc fracture dislocation. J Foot Ankle Surg.

[8] van Koperen PJ, de Jong VM, Luitse JS, Schepers T (2016). Functional outcomes after temporary bridging with locking plates in Lisfranc injuries. J Foot Ankle Surg.

[9] Moracia-Ochagavía I, Rodríguez-Merchán EC (2019). Lisfranc fracture-dislocations: current management. EFORT Open Rev.

[10] Brunet JA, Wiley JJ (1987). The late results of tarsometatarsal joint injuries. J Bone Joint Surg Br.

[11] (2013). Advanced trauma life support (ATLS®): the ninth edition. J Trauma Acute Care Surg.

[12] Gandek B, Ware JE, Aaronson NK (1998). Cross-validation of item selection and scoring for the SF-12 Health Survey in nine countries: results from the IQOLA Project. J Clin Epidemiol.

[13] Richter M (2020). EFAS Score Committee - Update 2020. Foot Ankle Surg.

[14] Holzer N, Salvo D, Marijnissen AC (2015). Radiographic evaluation of posttraumatic osteoarthritis of the ankle: the Kellgren-Lawrence scale is reliable and correlates with clinical symptoms. Osteoarthritis Cartilage.

[15] Buda M, Kink S, Stavenuiter R (2018). Reoperation rate differences between open reduction internal fixation and primary arthrodesis of Lisfranc injuries. Foot Ankle Int.

[16] Demirkale I, Tecimel O, Celik I, Kilicarslan K, Ocguder A, Dogan M (2013). The effect of the Tscherne injury pattern on the outcome of operatively treated Lisfranc fracture dislocations. Foot Ankle Surg.

[17] Dubois-Ferrière V, Lübbeke A, Chowdhary A, Stern R, Dominguez D, Assal M (2016). Clinical outcomes and development of symptomatic osteoarthritis 2 to 24 years after surgical treatment of tarsometatarsal joint complex injuries. J Bone Joint Surg Am.

[18] Eceviz E, Çevik HB, Öztürk O, Özen T, Çolak TK, Çolak İ, Polat MG (2021). Pedobarographic, clinic, and radiologic evaluation after surgically treated lisfranc injury. J Invest Surg.

[19] García-Renedo RJ, Carranza-Bencano A, Busta-Vallina B, Ortiz-Segura J, Plaza-García S, Gómez-del Alamo G (2012). [Long-term results of the treatment of Lisfranc fracture dislocation]. Acta Ortop Mex.

[20] Kuo RS, Tejwani NC, Digiovanni CW, Holt SK, Benirschke SK, Hansen ST Jr, Sangeorzan BJ (2000). Outcome after open reduction and internal fixation of Lisfranc joint injuries. J Bone Joint Surg Am.

[21] MacMahon A, Kim P, Levine DS (2016). Return to sports and physical activities after primary partial arthrodesis for lisfranc injuries in young patients. Foot Ankle Int.

[22] Marín-Peña OR, Viloria Recio F, Sanz Gómez T, Larrainzar Garijo R (2012). Fourteen years follow up after Lisfranc fracture-dislocation: functional and radiological results. Injury.

[23] Miswan MF, Singh VA, Yasin NF (2011). Outcome of surgically treated Lisfranc injury: a review of 34 cases. Ulus Travma Acil Cerrahi Derg.

[24] Nithyananth M, Boopalan PR, Titus VT, Sundararaj GD, Lee VN (2011). Long-term outcome of high-energy open Lisfranc injuries: a retrospective study. J Trauma.

[25] Poulsen M, Stødle AH, Nordsletten L, Röhrl SM (2023). Does temporary bridge plate fixation preserve joint motion after an unstable Lisfranc injury?. Foot Ankle Surg.

[26] Prasla SJ, Jiang SF, Pollard JD, Weintraub MR, Edlinger JP (2024). Mid-term incidence of tarsometatarsal joint arthrodesis following open reduction with internal fixation (ORIF) of Lisfranc injuries. J Foot Ankle Surg.

[27] Walley KC, Semaan DJ, Shah R, Robbins C, Walton DM, Holmes JR, Talusan PG (2021). Long-term follow-up of Lisfranc injuries treated with open reduction internal fixation patient-reported outcomes. Foot Ankle Orthop.

[28] Walsh A, Kasture S, Sugathan H, Dalal R (2023). Long term results and patient reported outcome measures following lisfranc injuries treated with memory staple fixation. Foot (Edinb).

[29] Pinsker E, Daniels TR (2011). AOFAS position statement regarding the future of the AOFAS Clinical Rating Systems. Foot Ankle Int.

[30] Larsen P, Rathleff MS, Roos EM, Elsoe R (2023). Foot and Ankle Outcome Score (FAOS): reference values from a national representative sample. Foot Ankle Orthop.

